# Effects of Fermented Palm Kernel Cake on Growth Performance, Serum Indices, and Rumen Microbiota in Growing–Fattening Beef Cattle

**DOI:** 10.3390/ani16142136

**Published:** 2026-07-09

**Authors:** Jie Shen, Duo Gao, Xiaodong Li, Ye Liu, Lei Wang, Junyan Wang, Miaomiao Xie, Yuan Xu, Kaijun Guo

**Affiliations:** 1 College of Animal Science and Technology, Beijing University of Agriculture, Beijing 102206, China; 18838270935@163.com (J.S.); godo0910@163.com (D.G.); 17853562667@163.com (Y.L.); 15810845801@163.com (L.W.); 15064828900@163.com (J.W.); xiemiaomiao11@163.com (M.X.); asdfcvbn990@163.com (Y.X.); 2 Beijing Doudian Hengsheng Animal Husbandry Center, Beijing 102402, China; li_xd68@163.com

**Keywords:** Angus cattle, fermented palm kernel cake, growth performance, serum indices, rumen microbiota

## Abstract

(1) Background: This study investigated the effects of fermented palm kernel cake (FPKC) on growth performance, serum indices, rumen fermentation and microbiota in growing–fattening beef cattle. (2) Methods: Forty-eight Angus cattle (504.50 ± 52.45 kg) were randomly divided into four groups (*n* = 12). The control group was fed a basal diet, while treatment groups were fed diets with 10%, 20% and 30% FPKC replacing corn and soybean meal concentrate. The experiment included a 14-day adaptation period and a 56-day formal experimental phase. (3) Results: Compared with the control, 20% FPKC significantly increased average daily gain and decreased the feed-to-gain ratio (*p* < 0.05). All FPKC groups had lower serum alanine aminotransferase (ALT) (*p* < 0.05). The 20% and 30% FPKC groups showed higher IgA and T-AOC, and lower MDA (*p* < 0.05) and highly significantly lower IL-1β. All FPKC groups had higher ruminal propionic acid (*p* < 0.05). In particular, 20% FPKC modified rumen microbial structure and increased the relative abundance of *Succinivibrionaceae_UCG-002* (*p* < 0.05). (4) Conclusions: Replacing part of concentrate with 20% FPKC (9.1% inclusion in the total diet) improved growth performance, enhanced immune and antioxidant function in growing–fattening beef cattle, optimized rumen fermentation, and increased the relative abundance of beneficial bacteria including *Succinivibrionaceae_UCG-002*.

## Simple Summary

Palm kernel cake, an agricultural by-product with rich nutrients, is a highly potential alternative feed ingredient for ruminant diets. Nevertheless, its nutritional value is restricted by anti-nutritional factors, which limits the application of palm kernel cake in ruminants. Microbial fermentation technology can improve feed quality and enhance the utilization efficiency of palm kernel cake in animals. However, the optimal dietary inclusion rate of fermented palm kernel cake (FPKC) in beef cattle remains unclear, and studies on beef cattle rumen fermentation and microbial modification are still insufficient and unsystematic. Therefore, this experiment was conducted to investigate the effects of replacing corn–soybean meal-based concentrates with different proportions of FPKC on growth performance, serum indices and ruminal microbiota in growing–fattening beef cattle. The results showed that replacing part of concentrates with 20% FPKC (9.1% dietary level) could improve growth performance, enhance immunity and antioxidant capacity, optimize ruminal fermentation patterns, and increase the relative abundance of *Succinivibrionaceae_UCG-002*, and thereby improve rumen microbial community structure. In summary, this study confirmed the feasibility of using FPKC as an alternative feed for growing–fattening beef cattle, which could provide practical references for beef cattle production.

## 1. Introduction

In recent years, with the annual expansion of farm scales, the animal husbandry industry has been confronted with enormous challenges in securing sustainable and economical feed resources. Traditional feed ingredients, such as corn and soybean meal, have experienced unstable supply and continuous cost increases, which become a core bottleneck restricting the high-quality development of animal husbandry [1]. Against this background, replacing traditional feed ingredients with low-cost agricultural and industrial by-products has become an important strategy. Palm kernel cake (PKC) is a by-product of palm oil extraction, which is low-cost and available in abundant supply, reaching more than 10 million tons annually worldwide for livestock feed [2]. In addition, palm kernel cake boasts advantages including rich crude protein content and abundant mineral elements [3], making it a popular alternative feed ingredient in animal production at present. However, previous studies have demonstrated contradictory results. Some studies showed that supplementation of palm kernel cake could exert negative effects on the average daily gain and nutrient digestibility of beef cattle, whereas others indicated that palm kernel cake significantly reduced feed intake without impairing cattle growth performance [4,5]. This discrepancy is mainly associated with its high content of non-starch polysaccharides (NSPs), among which β-mannan accounts for up to 78% [6]. β-mannan can encapsulate protein in the dense cell wall matrix, hindering nutrient absorption and digestion [7]. To overcome these limitations, microbial fermentation technology has become an effective approach to enhance the nutritional value of PKC. Many studies have demonstrated that microbial fermentation could effectively reduce its cellulose content, degrade anti-nutritional factors, and improve the overall nutritional value of palm kernel cake [8]. Specifically, *Aspergillus niger* produces polysaccharide hydrolases that degrade anti-nutritional factors in feed; *Saccharomyces cerevisiae* produces amylase and cellulase to break down cellulose; and *Lactobacillus plantarum* utilizes carbohydrates to improve feed nutritional value [9,10,11]. Huang et al. [12] found that fermented palm kernel cake replacing 40% wheat bran improved growth performance, feed utilization, and intestinal immunity in tilapia. Pin et al. [13] confirmed that fermented palm kernel cake replacing 75% soybean meal increased the apparent digestibility of dry matter, crude protein, neutral detergent fiber and acid detergent fiber in crossbred goats.

However, current studies on fermented palm kernel cake in beef cattle mostly focus on its application in Simmental cattle. There are few studies about systematic links among growth performance, rumen fermentation, rumen microbiota and immune or antioxidant functions, and the optimal dietary replacement level of fermented palm kernel cake for Angus beef cattle remains unclear. Therefore, this experiment aimed to investigate the effects of fermented palm kernel cake (FPKC) on growth performance, serum indices, rumen fermentation parameters, and the rumen microbiota of growing–fattening female cattle in order to determine the optimal supplementation level of FPKC as a replacement for part of the corn and soybean meal-based concentrate in the diet. This study is expected to provide comprehensive scientific evidence for the application of FPKC in ruminant production.

## 2. Materials and Methods

### 2.1. Preparation of Fermented Palm Kernel Cake

The palm kernel cake used in this experiment was provided by Qingdao Nanyang Jinmu International Trading Company. *Aspergillus niger*, *Saccharomyces cerevisiae*, and *Lactobacillus plantarum* were originally obtained from the China National Institute of Standardization and maintained in the Intelligent Breeding Laboratory of Beijing University of Agriculture. Prior to fermentation, *Aspergillus niger* was inoculated on potato dextrose agar medium and activated at 30 °C for 72 h; *Saccharomyces cerevisiae* was inoculated on yeast extract peptone dextrose medium and activated at 30 °C for 48 h; *Lactiplantibacillus plantarum* was inoculated on de Man, Rogosa and Sharpe medium and activated at 37 °C for 24 h. According to previous studies conducted in our own laboratory, the optimal fermentation conditions for PKC were as follows: palm kernel cake and wheat bran mixed at a ratio of 4:1, and a mixed bacterial solution containing 2% *Aspergillus niger* (≥1 × 10^7^ CFU/g), *Saccharomyces cerevisiae* (≥1 × 10^8^ CFU/g), and *Lactobacillus plantarum* (≥1 × 10^8^ CFU/g) at a ratio of 1:1:1. The water content of the mixture was adjusted to 50%, and the mixture was placed in plastic fermentation bags with one-way exhaust valves and fermented at ambient temperature for 4 days before use. Nutritional composition of PKC and FPKC was compared to characterize fermentation-induced nutrient variation and provide basic data for subsequent feeding experiment analysis. For statistical analysis of nutritional indicators, three replicate samples were prepared for both PKC and FPKC.

### 2.2. Animals, Diets, and Experimental Design

This experiment was conducted from April to July 2025 at Beijing Doudian Hengsheng Animal Husbandry Center. A total of 48 healthy 15–16-month Angus female cattle with an average body weight of 504.50 ± 52.45 kg were randomly assigned to 4 groups (*n* = 12), with 3 cattle per pen and 4 pens per group. The control group (CON) was fed a basal diet with corn and soybean meal-based concentrate, while the three treatment groups (10% FPKC group, 20% FPKC group and 30% FPKC group) were fed diets in which 10%, 20% and 30% of the concentrate were replaced by FPKC. The TMR diet was formulated with reference to China Beef Cattle Feeding Standard (NY/T 815-2004) [14], and its composition and nutrient levels are shown in Table 1 The experiment lasted for 70 days, including a 14-day pre-feeding period and a 56-day formal trial period. The cattle were fed *ad libitum* at 7:00 a.m. and 4:00 p.m. daily with free access to water.

### 2.3. Sample Collection and Index Determination

#### 2.3.1. Chemical Analysis

The nutritional components of PKC, FPKC, and experimental diets were analyzed by the following methods. Dry matter (DM; Method 934.01), crude protein (CP; Method 954.01), ether extract (EE; Method 920.39), crude ash (Ash; Method 942.05), starch (Method 996.11), calcium (Ca; Method 968.08), and phosphorus (P; Method 946.06) were determined in accordance with Association of Official Analytical Chemists (AOAC) standard procedures [15]. Neutral detergent fiber (NDF) and acid detergent fiber (ADF) were measured using the method described by Van Soest et al. [16].

#### 2.3.2. Growth Performance

The body weight of each cattle was recorded before morning feeding on the first and last days of the formal trial period. During the 26th–30th day, total feed offered and residual feed per pen were recorded daily. The average daily gain (ADG), dry matter intake (DMI), and feed-to-gain ratio were calculated.

Average daily gain (ADG, kg) = (Final body weight − Initial body weight)/Feeding days in the formal trial period

Dry matter intake (DMI, kg) = (Feed offered in DM − residual feed in DM)/(Number of beef cattle per group)

Feed-to-gain ratio (F/G) = DMI/ADG

#### 2.3.3. Serum Indices

On day 56 of the formal trial, before morning feeding, 10 mL of blood was collected from the caudal vein of each experimental cattle. Samples were centrifuged at 3500 r/min for 10 min at 4 °C. Serum was separated and stored at −20 °C for analysis of biochemical, immune and antioxidant indices. Serum biochemical indices included alanine aminotransferase (ALT), aspartate aminotransferase (AST), alkaline phosphatase (ALP), total protein (TP), albumin (ALB), glucose (GLU), urea (UREA), and creatinine (CREA); these indices were measured using an automatic biochemical analyzer (TBA-120FR, Japan). Serum immune indices consisted of tumor necrosis factor-α (TNF-α), interleukin-1β (IL-1β), immunoglobulin A (IgA), and immunoglobulin M (IgM). Serum antioxidant indices included total superoxide dismutase (SOD), malondialdehyde (MDA), total antioxidant capacity (T-AOC), and catalase (CAT). The immune and antioxidant indices were assayed strictly following the manufacturer’s instructions provided with the commercial kits (Nanjing Jiancheng Bioengineering Institute, Nanjing, China).

#### 2.3.4. Rumen Fermentation Parameters

On day 56 of the formal trial, before morning feeding, rumen fluid was collected from all experimental cattle in each group via the oral cavity using a rumen fluid collection tube. The initial 50 mL of the collected sample was discarded to prevent contamination by saliva. Subsequently, the remaining rumen fluid was filtered through four layers of gauze, and its pH value was immediately measured using a portable pH meter (Professional pH Test Pen DLX-GM761, Delixi Electric, Wenzhou, China). The filtered rumen fluid was then divided into two portions: one portion was used for the determination of rumen fermentation parameters, and the other portion was used for 16S rRNA gene sequencing. Among the fermentation parameters, ammonia nitrogen (NH_3_-N) was assayed following the method described by Li et al. [17], while microbial crude protein (MCP) was determined according to the protocol established by Fan et al. [18]. Volatile fatty acids (VFAs) were analyzed using an ultra-performance liquid chromatography (UPLC) system (ACQUITY UPLC, Waters Technology, Shanghai, China). The chromatographic conditions were set as follows: a C18-T3 column (50 mm × 2.1 mm × 1.8 μm) was employed; the mobile phase consisted of 10% acetonitrile and 90% aqueous solution containing 0.05% phosphoric acid; the flow rate was maintained at 0.7 mL/min; and the total detection runtime was 6 min.

#### 2.3.5. 16S rRNA Gene Sequencing and Data Processing

Four cattle were randomly selected from the four replicate pens of each treatment for rumen fluid sampling and 16S rRNA gene sequencing. Total DNA was extracted using the OMEGA-soil DNA kit (Omega Bio-tek, Norcross, GA, USA). DNA integrity was verified by 1% agarose gel electrophoresis, and DNA concentration and purity were determined using a NanoDrop2000 (Thermo Scientific, Waltham, MA, USA). The V3–V4 region of the 16S rRNA gene was amplified with barcode-containing primers 338F/806R. PCR products were recovered by 2% agarose gel electrophoresis, purified with a PCR Clean-Up kit (Shanghai Majorbio Yuhua Biomedical Technology Co., Ltd., Shanghai, China), and quantified using the QuantiFluor™-ST system (Promega, Madison, WI, USA). Sequencing libraries were constructed using the NEXTFLEX Rapid DNA-Seq kit (Revvity, Austin, TX, USA) and sequenced on the Illumina Nextseq 2000 platform (Shanghai Majorbio Bio-pharm Technology Co., Ltd., Shanghai, China). Raw reads were quality-controlled using fastp, merged with FLASH, and clustered into operational taxonomic units (OTUs) at 97% similarity using UPARSE (V11) with chimeras removed. Taxonomic annotation was performed using the RDP Classifier against the Silva 16S rRNA database, and microbial community composition was analyzed at all taxonomic levels.

Data analysis was conducted on the Majorbio Cloud Platform (https://cloud.majorbio.com) with the following procedures: Alpha diversity indices were calculated using the mothur software (Version 1.48.0), and the Wilcoxon rank-sum test was used to analyze intergroup differences in Alpha diversity. Principal Coordinate Analysis (PCoA) based on the Bray–Curtis distance algorithm was performed to examine the similarities or differences in microbial community structure among samples. Linear discriminant analysis Effect Size (LEfSe) analysis was conducted with the thresholds of LDA > 3 and *p* < 0.05 was conducted to identify bacterial taxa with significantly different abundances from the phylum to genus levels among CON and three treatments.

### 2.4. Statistical Analysis

The experimental data were compiled and initially organized using Excel 2019. Data on growth performance, serum indices and rumen fermentation parameters were analyzed using one-way analysis of variance (ANOVA) in the SPSS 27.0 software. Dry matter intake, feed-to-gain ratio and average daily gain were analyzed by pen, whereas blood indicators and rumen fermentation parameters were analyzed per animal. All data underwent multiple comparisons using Duncan’s method. The significance thresholds were set as follows: *p* < 0.01 indicated a highly significant difference, *p* < 0.05 indicated a significant difference, and 0.05 ≤ *p* < 0.1 indicated a tendency toward significant difference. The results were expressed as mean ± standard error of the mean (*SEM*).

## 3. Results

### 3.1. Nutrient Composition of Palm Kernel Cake Before and After Fermentation

The nutrient contents of PKC before and after fermentation are shown in Table 2. It can be seen that after fermentation, the CP content of palm kernel cake highly significantly increased, while the ADF, NDF, and EE contents highly significantly decreased (*p* < 0.01).

### 3.2. Growth Performance

As shown in Table 3, compared with the CON group, only the 20% FPKC group significantly increased in ADG and significantly decreased in F/G (*p* < 0.05). In addition, both the 20% FPKC and 30% FPKC groups had highly significantly higher DMI than the CON group (*p* < 0.01).

### 3.3. Serum Indices

As shown in Table 4, compared with CON, the ALT content in all treatment groups was significantly decreased (*p* < 0.05). In comparison with CON and 10% FPKC groups, 20% FPKC and 30% FPKC groups exhibited highly significantly increased GLU content (*p* < 0.01), significantly increased IgA and T-AOC contents (*p* < 0.05), significantly decreased UREA content (*p* < 0.05), and highly significantly decreased IL-1β content (*p* < 0.01). Compared with CON and the 10% FPKC groups, the 20% FPKC group showed a significant decrease in MDA content and a significant increase in IgM content (*p* < 0.05). Additionally, the TNF-α content in the 30% FPKC group was significantly lower than that in CON and the 10% FPKC group (*p* < 0.05).

### 3.4. Rumen Fermentation Parameters

As shown in Table 5, compared with CON, all treatment groups highly significantly increased the propionic acid content (*p* < 0.01). Compared with CON, the 20% FPKC group significantly increased the MCP content and significantly decreased the acetate/propionate ratio (*p* < 0.05).

### 3.5. Rumen Microbiota

#### 3.5.1. Analysis of Rumen Microbial OTUs and Diversity

**Figure 1:**
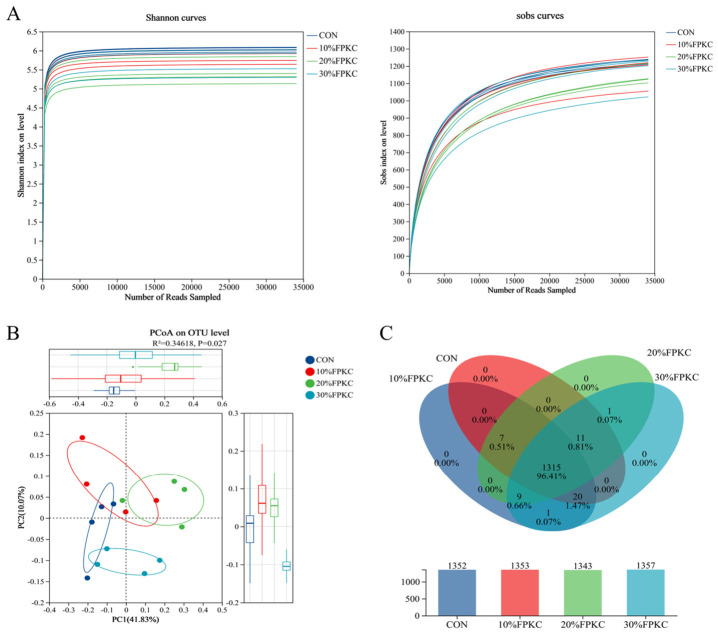
Effects of FPKC on rumen microbial composition. (**A**) Rarefaction curves. (**B**) Principal Coordinate Analysis (PCoA). (**C**) Venn diagram comparison of OTUs between CON and three treatment groups.

After quality control and primer removal, a total of 808,924 optimized valid sequences were obtained, with an average length of 418 bp. Clustering of the valid sequences at a similarity level of >97% yielded 1364 OTUs, among which 1315 OTUs were shared by all groups, accounting for 96.41% of the total. Notably, no unique OTU was detected in any treatment group, revealing high consistency in microbial community composition among groups (Figure 1C). The rarefaction curves of Sobs and Shannon indices for rumen fluid samples (Figure 1A) exhibited clear asymptotes, indicating reliable sequencing depth sufficient for microbial diversity analysis. PCoA analysis at the OTU level (Figure 1B) revealed significant differences in microbial community structure among groups (*p* < 0.05), with the first and second axes explaining 10.07% and 41.83% of the total variation, respectively. As shown in Table S1, replacing part of the concentrate with FPKC did not significantly affect the rumen bacterial Alpha diversity indices, including Sobs, ACE, Shannon, Simpson, and Chao1.

#### 3.5.2. Relative Abundance of Rumen Microbiota at the Phylum Level

**Figure 2:**
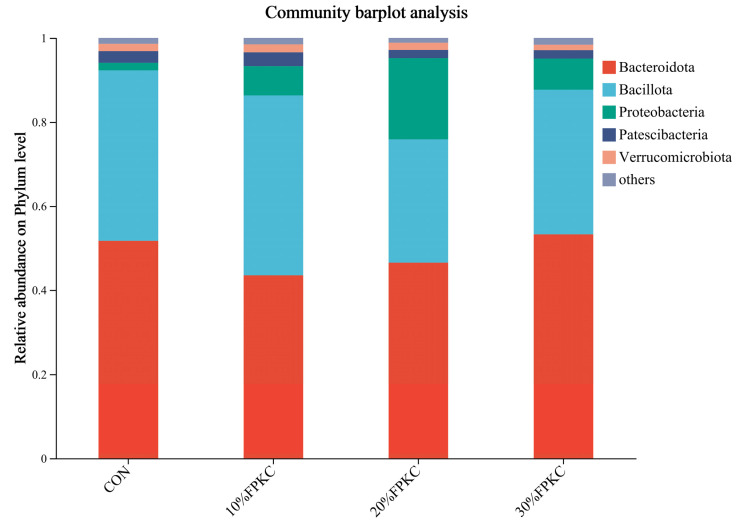
Relative abundance of rumen microbiota at the phylum level among different groups.

**Figure 3:**
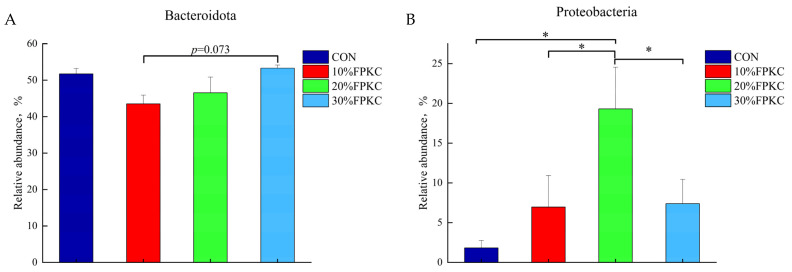
Relative abundance of significantly different rumen microbiota species (top 15) at the phylum level among different groups. (**A**) Relative abundance of Bacteroidota. (**B**) Relative abundance of Proteobacteria. * indicates significant differences (*p* < 0.05).

As shown in Figure 2, a total of six bacterial phyla were identified, among which five phyla had a relative abundance exceeding 1%. The dominant microbial taxa included Bacteroidota, Bacillota, Proteobacteria, and Patescibacteria. To further clarify intergroup differences in the phylum-level microbiota, ANOVA was performed on all dominant taxa, with detailed results shown in Table S2. Significantly different taxa were visualized using Origin software (Version OriginPro 2024 SR1, 10.1.0.178), with the results presented in Figure 3. As shown in Figure 3A, with the increase in the supplementation ratio of FPKC, the relative abundance of Bacteroidota in the rumen microflora also increased continuously, and the 30% FPKC group showed a tendency for significant increase compared with the 10% FPKC group (*p* = 0.07). Notably, the relative abundance of Proteobacteria in the 20% FPKC group was significantly higher than that in the other groups (*p* < 0.05) (Figure 3B).

#### 3.5.3. Relative Abundance of Rumen Microbiota at the Genus Level

**Figure 4:**
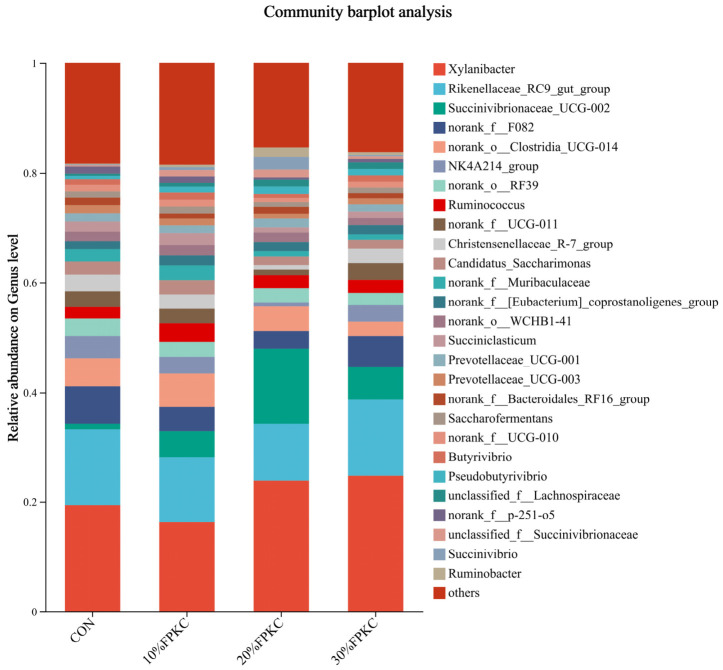
Relative abundance of rumen microbiota at the genus level among different groups.

**Figure 5:**
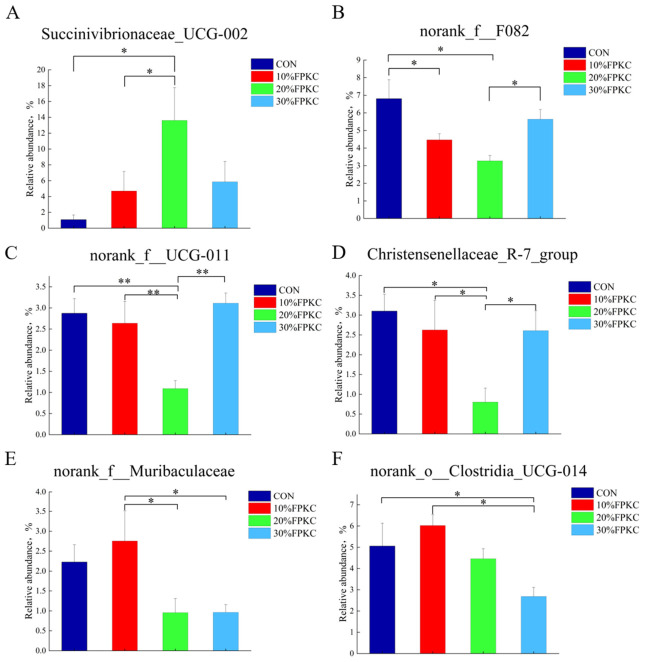
Relative abundance of significantly different rumen microbiota species (top 15) at the genus level among different groups. (**A**) Relative abundance of *Succinivibrionaceae_UCG-002*. (**B**) Relative abundance of *norank_f__F082*. (**C**) Relative abundance of *norank_f__UCG-011*. (**D**) Relative abundance of *Christensenellaceae_R-7_group*. (**E**) Relative abundance of *norank_f__Muribaculaceae*. (**F**) Relative abundance of *norank_o__Clostridia_UCG_014*. * indicates significant differences (*p* < 0.05), ** indicates highly significant differences (*p* < 0.01).

As shown in Figure 4, a total of 38 bacterial genera were identified, among which 27 genera had a relative abundance exceeding 1%. The dominant genera included *Xylanibacter*, *Rikenellaceae_RC9_gut_group*, *Succinivibrionaceae_UCG-002*, *norank_f__F082*, and *norank_o__Clostridia_UCG_014*. To further clarify intergroup differences in the genus-level microbiota, ANOVA was performed on all dominant taxa, with detailed results shown in Table S3. Significantly different taxa were visualized using Origin software, with the results presented in Figure 5. As shown in Figure 5E, the relative abundance of *norank_f__Muribaculaceae* in the 10% FPKC group was significantly higher than that in the 20% FPKC and 30% FPKC groups (*p* < 0.05). The relative abundance of *norank_o__Clostridia_UCG_014* in the 30% FPKC group was significantly lower than that in CON (*p* < 0.05) (Figure 5F). In the 20% FPKC group, the relative abundance of *Succinivibrionaceae_UCG-002* was significantly higher than that in CON, while the relative abundances of norank_f__UCG-011 was highly significantly lower (*p* < 0.01), and the relative abundances of *Christensenellaceae_R-7_group* and *norank_f__F082* were significantly lower than those in CON (*p* < 0.05) (Figure 5A–D).

#### 3.5.4. LEfSe Analysis of Differential Rumen Microbiota

**Figure 6:**
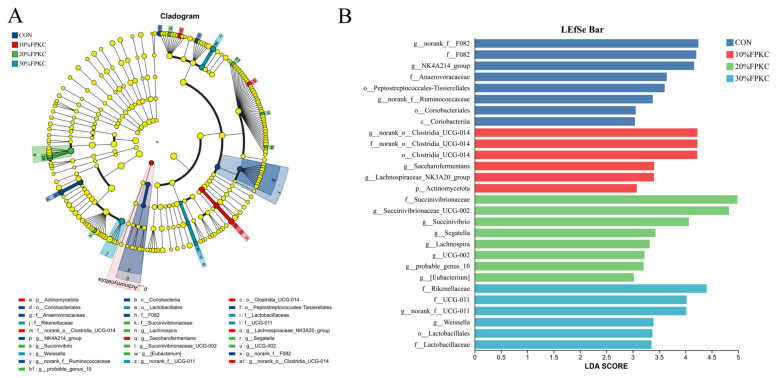
LEfSe analysis of differential rumen microbiota among the four groups (*p* < 0.05, LDA > 3.0). (**A**) Nodes of different colors represent the differential microbial taxa significantly enriched in the corresponding groups; pale yellow nodes represent the taxa with no significant differences among groups. (**B**) The bar chart shows the LDA effect sizes of differential microbial taxa, with larger values indicating a greater impact of species abundance on intergroup differences.

Differential microbiota were analyzed using LEfSe, and a total of 28 statistically differential species were detected (Figure 6A,B). Specifically, the number of differential taxa identified was eight in the CON group, six in the 10% FPKC group, eight in the 20% FPKC group, and six in the 30% FPKC group. Compared with the control group, the most significantly different and enriched microorganism in the 10% FPKC group was *g__norank_o__Clostridia_UCG-014* (*p* = 0.041); in the 20% FPKC group, it was f__Succinivibrionaceae (*p* = 0.045); and in the 30% FPKC group, it was f__Rikenellaceae (*p* = 0.028).

#### 3.5.5. Spearman Correlation Analysis Between Rumen Fermentation Parameters and Rumen Microbiota

**Figure 7:**
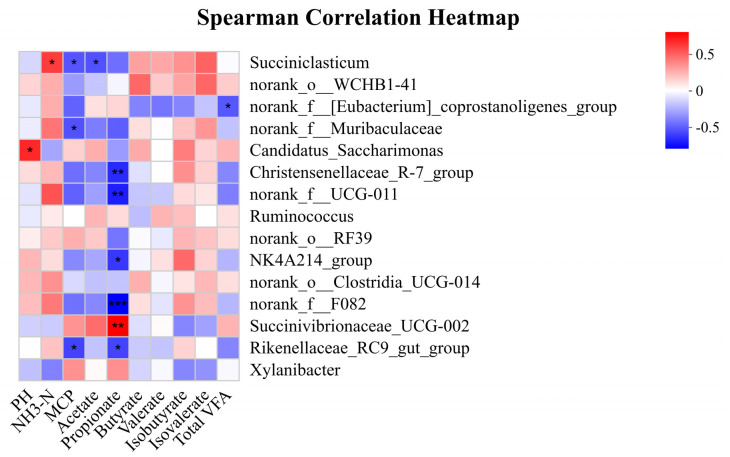
Spearman analysis of top 15 rumen microbiota abundances (genus level) and rumen fermentation parameters. Red indicates positive correlation, blue indicates negative correlation, and the darker the color, the stronger the correlation. Significance levels are marked as follows: 0.01 < *p* ≤ 0.05 (*), 0.001 < *p* ≤ 0.01 (**), *p* ≤ 0.001 (***).

It can be seen from Figure 7 that 10 microbial genera showed significant correlations at the genus level. Among them, total volatile fatty acids (TVFAs) were significantly negatively correlated with *norank_f__[Eubacterium]_coprostanoligenes_group* (*p* < 0.05), and rumen pH exhibited a highly significant positive correlation with *Candidatus_Saccharimonas* (*p* < 0.01). *Succiniclasticum* abundance was significantly negatively related to MCP and acetate (*p* < 0.05) and positively associated with NH_3_-N (*p* < 0.05). MCP showed significant negative correlations with *norank_f__Muribaculaceae* and *Rikenellaceae_RC9_gut_group* (*p* < 0.05). Propionate had significant negative correlations with *Rikenellaceae_RC9_gut_group* and *NK4A214_group* (*p* < 0.05), highly significant negative correlations with *norank_f__UCG-011*, *norank_f__F082* and *Christensenellaceae_R-7_group* (*p* < 0.01), and a highly significant positive correlation with *Succinivibrionaceae_UCG-002* (*p* < 0.01).

## 4. Discussion

### 4.1. Effects of FPKC on Growth Performance of Growing–Fattening Beef Cattle

In the development of animal husbandry, the growth performance of animals affects the economic benefits of the industry. ADG and F/G are key parameters for evaluating the growth performance of growing–fattening beef cattle [19]. In this experiment, *Aspergillus niger*, *Saccharomyces cerevisiae* and *Lactobacillus plantarum* were used for the preparation of FPKC. The fermentation not only reduced the contents of NDF and ADF, but also improved feed palatability and enhanced the digestive and absorptive capacity of growing–fattening beef cattle, resulting in an increase in ADG and a decrease in F/G. Among all the experimental groups, the 20% FPKC group exhibited significant effects in ADG, whereas the 10% FPKC and 30% FPKC groups showed no significant differences. This phenomenon might be associated with the supplementation ratio, as an optimal proportion enables FPKC to achieve the best performance when replacing part of the concentrates in the diet. DMI of growing–fattening beef cattle in the 20% FPKC and 30% FPKC groups was significantly increased. This could be explained by the fact that *Lactobacillus plantarum* has the ability to decompose cellobiose, and the fermentation process can produce an acid flavor, thereby enhancing the feed intake of animals [20]. Overall, FPKC did not exert any negative effects on the growth performance of growing–fattening beef cattle. This finding is consistent with that of Pin et al. [13], who reported that substituting soybean meal with fermented palm kernel cake had no adverse effects on the growth performance of goats. Similarly, Mi et al. [21] used *Aspergillus niger* and β-mannanase to ferment palm kernel cake for beef cattle and observed no significant changes in growth performance.

### 4.2. Effects of FPKC on Serum Indices of Growing–Fattening Beef Cattle

Serum indices are important markers of dietary metabolism in animals and can reflect the metabolic status of nutrients in the body. By detecting relevant indicators in animal serum, we can determine whether the animals are in a good physiological state. AST and ALT are important transaminases in animals, which can reflect the health status of the liver and heart function; meanwhile, they also serve as biomarkers of metabolic stress [22]. In this study, the ALT level in the experimental groups decreased significantly, indicating that FPKC could enhance hepatic metabolic function and exert positive effects on maintaining the structural integrity of hepatocytes. GLU is not only the main energy source for cells but can also reflect the energy metabolic status, and its content changes are associated with energy consumption and supply [23]. In this study, the GLU levels in the 20% FPKC and 30% FPKC groups increased significantly, which may indicate that with the increase in the supplementation ratio of FPKC, the body’s capacity for energy digestion and supply was higher. This can also explain why the DMI was significantly increased in the 20% FPKC and 30% FPKC groups. Urea can reflect the body’s ability to catabolize proteins. Studies have shown that there is a significant negative correlation between blood urea content and the protein utilization rate; a decrease in urea content indicates an improvement in protein utilization [24]. In this experiment, the urea levels in the 20% FPKC and 30% FPKC groups decreased, suggesting that their protein utilization efficiency was enhanced. In addition, there were no significant differences in TP and ALB levels among all treatment groups, which indicates that FPKC does not exert adverse effects on protein synthesis and metabolism.

IgA, IgM, and IgG are important immunoglobulins in animals; they are antibodies produced by lymphocytes of the body’s immune system through a series of transformations, and their levels are key indicators reflecting the immune status of the body [25]. IL-1β and TNF-α are proinflammatory cytokines that participate in inflammatory responses [26]. In this study, the 20% FPKC and 30% FPKC groups significantly decreased IL-1β and TNF-α levels, and significantly increased IgA levels, indicating that FPKC can improve the immune capacity and resistance to adverse external environments of growing–fattening beef cattle to a certain extent. On the one hand, this might be attributed to the fact that FPKC converts macromolecules into small molecules and promotes the release of bioactive components, which can enhance nonspecific immunity to a certain degree [27]. On the other hand, it may be because the probiotics in FPKC can stimulate the lymphoid tissues of the body and promote the secretion of immunoglobulins, thereby activating the systemic immune system, and ultimately enhancing the body’s immunity [28].

T-AOC reflects the comprehensive antioxidant level generated by antioxidant substances and antioxidant enzymes in the body [29]. MDA is the final product of lipid peroxidation, reflecting the degree of tissue peroxidation damage [30]. SOD and CAT can alleviate oxidative stress; among them, SOD can directly reflect the body’s ability to scavenge free radicals [31]. In this study, the 20% FPKC and 30% FPKC groups significantly increased T-AOC levels and significantly decreased MDA content, indicating that FPKC helps enhance the antioxidant capacity of growing–fattening beef cattle. This might be attributed to the production of various metabolites during the fermentation of FPKC, including phenolic compounds and flavonoids. These biomolecules have strong antioxidant properties, which can scavenge free radicals and reduce oxidative damage [32].

### 4.3. Effects of FPKC on Rumen Fermentation Parameters of Growing–Fattening Beef Cattle

The pH of rumen fluid is a key indicator reflecting the rumen fermentation level. A pH range of 6.7 ± 0.5 can maintain stable cellulolytic activity [33]. In this study, the rumen fluid pH ranged from 6.45 to 6.69, all falling within this interval, indicating that FPKC can maintain normal rumen function without exerting adverse effects. Volatile fatty acids are produced by rumen fermentation and serve as the main energy source for ruminants. Among them, propionic acid is the primary substrate for gluconeogenesis in ruminants; an increase in propionic acid can prevent amino acids from decomposing into volatile fatty acids and carbon dioxide [34]. In this study, the 20% FPKC group showed a significant increase in propionic acid content and a significant decrease in the acetate-to-propionate ratio, which implied high feed utilization efficiency. This phenomenon may be attributed to microbial fermentation converting non-starch polysaccharides in palm kernel cake into soluble sugars, whose fermentation may increase propionate concentration in the rumen. This finding is consistent with the research results of Wang et al. [35], who used fermented rice straw to replace peanut vine in feeding Hu sheep and found that the 50% fermented rice straw replacement group significantly increased propionic acid content and significantly reduced the acetate-to-propionate ratio.

Ammonia is an important nitrogen source for the synthesis of MCP. Rumen microbiota can utilize NH_3_-N produced by degradation as a nitrogen source [36]. MCP provides 60–80% of the absorbable amino acids for ruminants, which is crucial for ruminant productivity. In this study, NH_3_-N concentrations in all groups were within the optimal range (5–30 mg/dL) [37], and the 20% FPKC group exhibited a significant increase in MCP content, indicating that FPKC improves the efficiency of rumen microbial utilization of dietary protein.

### 4.4. Effects of FPKC on Rumen Microbiota of Growing–Fattening Beef Cattle

The rumen is a complex ecosystem characterized by a dynamic balance between the host and its microbiota, and among the microbiota themselves [38]. The results of this study indicated that the experimental groups did not affect the richness and α-diversity of the rumen microbial community, suggesting that the components in FPKC had no impact on these indicators. Principal component analysis showed partial overlap between the 10% FPKC and 30% FPKC groups and CON, while the 20% FPKC group was completely separated from CON. This demonstrated that FPKC altered the rumen microbial community structure, with the 20% FPKC group showing the most pronounced changes.

At the phylum level, Bacteroidota, Bacillota, and Proteobacteria collectively constituted the dominant components of the rumen microbiota, accounting for over 90% of the total bacterial population in this study. This finding was highly consistent with the research conclusion of Paul et al. [39] stating “these three bacterial phyla are the main microbial groups in cattle and buffalo”. In this study, the Bacteroidota/Bacillota ratio was 1.29 for the control group, 1.10 for the 10% FPKC group, 1.66 for the 20% FPKC group and 1.62 for the 30% FPKC group. The 20% FPKC group presented the highest ratio, indicating a stronger capacity to improve feed efficiency and promote energy absorption in growing–fattening beef cattle [40]. Proteobacteria can utilize proteins and degrade carbohydrates to produce energy [41]; thus, an increase in Proteobacteria may have promoted protein metabolism to a certain extent. At the genus level, two taxa belonging to Bacteroidota, namely *norank_f__F082* and *norank_f__Muribaculaceae*, exhibited significant differences in relative abundance. The taxa with significant differences, including *norank_o__Clostridia_UCG-014*, *norank_f__UCG-011*, and *Christensenellaceae_R-7_group*, belonged to Firmicutes, while *Succinivibrionaceae_UCG-002* belonged to Proteobacteria. In this experiment, the relative abundance of *Succinivibrionaceae_UCG-002* in the 20% FPKC group increased significantly, whereas that of other genera decreased. This might be because *Succinivibrionaceae_UCG-002* was a dominant microbial group that occupied a leading position and thus inhibited the proliferation of other microbial groups. In addition, *Succinivibrionaceae_UCG-002* is a fiber-degrading bacterium that can decompose cellulose and cellobiose into succinic acid, participate in succinic acid production, and generate propionic acid via the succinic acid pathway [42]. This may also explain why the propionic acid content in the 20% FPKC group was significantly higher than that in CON. Meanwhile, Spearman correlation analysis showed that *Succinivibrionaceae_UCG-002* in the 20% FPKC group was extremely significantly positively correlated with ruminal propionate content. This result was consistent with previous findings in the present study, further confirming that this bacterium plays an important role in regulating ruminal propionate synthesis.

## 5. Conclusions

In summary, substituting 20% of corn, soybean meal, palm kernel cake and jujube powder-based concentrates with FPKC (9.1% inclusion in the total diet) significantly improved the growth performance of growing–fattening beef cattle, markedly reduced the contents of ALT and UREA, enhanced their antioxidant capacity and immune function, ameliorated rumen fermentation, regulated rumen microbiota community structure, and increased the relative abundance of *Succinivibrionaceae_UCG-002*. These findings provide fundamental data support for the application of FPKC in growing–fattening beef cattle production. Furthermore, these findings offer a feasible strategy for reducing the usage of corn and soybean meal via substitution.

## Figures and Tables

**Table 1:** Composition and nutrient levels of basal diets in control and treatment groups (% dry matter basis).

Items	Treatments ^1^			
	CON	10% FPKC	20% FPKC	30% FPKC
Corn	26.00	23.40	20.80	18.20
Soybean meal	7.50	6.75	6.00	5.25
Jujube powder	6.00	5.40	4.80	4.20
Palm kernel cake	6.00	5.40	4.80	4.20
Premix ^2^	2.00	2.00	2.00	2.00
NaHCO_3_	1.00	1.00	1.00	1.00
Limestone	1.00	1.00	1.00	1.00
NaCl	0.50	0.50	0.50	0.50
Corn silage	25.71	25.71	25.71	25.71
Rice straw	24.29	24.29	24.29	24.29
FPKC	0.00	4.55	9.10	13.65
Total	100.00	100.00	100.00	100.00
Nutrient levels (% of DM)				
NEmf/(MJ/kg) ^3^	5.14	5.22	5.35	5.39
DM (% As fed)	49.69	49.00	48.27	48.15
CP (% of DM)	9.63	9.50	9.70	9.80
EE (% of DM)	2.70	3.00	3.30	3.60
NDF (% of DM)	43.22	44.68	45.95	46.98
ADF (% of DM)	28.57	29.26	29.51	29.90
Starch (% of DM)	21.82	21.53	21.10	20.80
Ca (% of DM)	0.48	0.47	0.49	0.48
P (% of DM)	0.33	0.30	0.34	0.32

^1^ CON: control group, fed a basal diet; 10% FPKC, 10% fermented palm kernel cake replacing part of the concentrate; 20% FPKC, 20% fermented palm kernel cake replacing part of the concentrate; 30% FPKC, 30% fermented palm kernel cake replacing part of the concentrate. The same applies to the tables below. ^2^ Each kilogram of premix contains 150,000–450,000 IU of vitamin A acetate, 40,000–120,000 IU of vitamin D_3_, ≥400 mg of dl-α-tocopheryl acetate, 1000–3000 mg of manganese, 1000–5000 mg of iron, 1500–3700 mg of zinc, 250–750 mg of copper, 100–250 g of calcium, ≥300 g of total phosphorus, and 150–300 g of sodium chloride. ^3^ The comprehensive net energy was calculated with reference to NY/T 815-2004, and the other values were measured values.

**Table 2:** Nutrient composition of PKC before and after fermentation (% dry matter basis).

Items ^1^	PKC	FPKC	*SEM*	*p*-Value
CP	17.63	19.82	0.51	0.002
ADF	42.95	37.38	1.20	0.002
NDF	62.62	60.24	0.57	0.007
EE	7.91	6.28	0.37	<0.001
Ash	4.95	5.46	1.48	0.068

^1^ CP, crude protein; ADF, acid detergent fiber; NDF, neutral detergent fiber; EE, ether extract; PKC, palm kernel cake; FPKC, fermented palm kernel cake.

**Table 3:** Effects of FPKC on the growth performance of growing–fattening beef cattle.

Items ^1^	Treatments				*SEM*	*p*-Value		
	CON	10% FPKC	20% FPKC	30% FPKC		*Treatment Effect*	*Linear Effect*	*Quadratic Effect*
IBW, kg	503.86	506.29	502.00	505.86	9.910	0.999	0.985	0.999
FBW, kg	560.86	568.00	569.14	561.86	9.710	0.988	0.963	0.937
ADG, kg/d	1.02 ^b^	1.10 ^ab^	1.20 ^a^	1.00 ^b^	0.026	0.015	0.854	0.016
DMI ^2^, kg/d	11.89 ^C^	11.80 ^BC^	12.38 ^A^	11.96 ^B^	0.033	<0.001	0.006	0.001
F/G	11.66 ^ab^	10.73 ^bc^	10.32 ^c^	11.96 ^a^	0.224	0.021	0.805	0.010

a–c: Within a row, values without a common superscript differ significantly (*p* < 0.05). A–C: Within a row, there are highly significant differences (*p* < 0.01) between values without a common superscript. ^1^ IBW, initial body weight; FBW, final body weight; ADG, average daily gain; DMI, dry matter intake; F/G, feed-to-gain ratio. ^2^ DMI data were analyzed using repeated measures analysis, *p* (day) < 0.001, *p* (treatment × day) = 0.004.

**Table 4:** Effects of FPKC on serum biochemical, immune and antioxidant indices of growing–fattening beef cattle.

Items ^1^	Treatments				*SEM*	*p*-Value		
	CON	10% FPKC	20% FPKC	30% FPKC		*Treatment Effect*	*Linear Effect*	*Quadratic Effect*
ALT, U/L	38.67 ^a^	32.67 ^b^	33.00 ^b^	29.33 ^b^	1.228	0.023	0.004	0.017
AST, U/L	86.33	77.67	79.33	76.00	1.999	0.302	0.099	0.218
TP, g/L	74.43	72.28	73.18	74.03	0.499	0.486	0.918	0.363
ALB, g/L	38.33	37.83	38.35	37.33	0.268	0.472	0.261	0.505
GLU, mmol/L	3.68 ^B^	3.74 ^B^	4.34 ^A^	4.33 ^A^	0.106	0.004	0.001	0.008
UREA, mmol/L	3.05 ^a^	3.05 ^a^	2.53 ^b^	2.58 ^b^	0.908	0.032	0.010	0.042
CREA, umol/L	109.50	107.00	106.00	105.00	0.944	0.361	0.068	0.187
TNF-α, pg/mL	13.29 ^ab^	15.66 ^a^	10.62 ^bc^	8.88 ^c^	0.920	0.014	0.017	0.025
IL-1β, pg/mL	11.42 ^A^	11.28 ^A^	6.15 ^B^	7.26 ^B^	0.739	0.003	0.003	0.013
IgA, g/L	0.58 ^b^	0.57 ^b^	0.71 ^a^	0.73 ^a^	0.028	0.030	0.007	0.032
IgM, g/L	2.10 ^b^	2.11 ^b^	2.58 ^a^	2.51 ^ab^	0.085	0.042	0.016	0.061
IgG, g/L	9.51	9.24	9.70	9.67	0.248	0.925	0.666	0.894
SOD, U/mL	81.59	77.89	82.44	83.34	1.489	0.632	0.482	0.600
MDA, nmol/mL	4.67 ^a^	4.60 ^ab^	3.98 ^c^	4.16 ^bc^	0.102	0.019	0.010	0.031
T-AOC, U/mL	6.06 ^b^	5.65 ^b^	6.96 ^a^	6.98 ^a^	0.208	0.019	0.020	0.063
CAT, U/mL	12.16	12.51	12.08	12.56	0.198	0.826	0.684	0.915

a–c: Within a row, values without a common superscript differ significantly (*p* < 0.05). A,B: Within a row, there are highly significant differences (*p* < 0.01) between values without a common superscript. ^1^ ALT, alanine aminotransferase; AST, aspartate aminotransferase; TP, total protein; ALB, albumin; GLU, glucose; UREA, urea; CREA, creatinine; TNF-α, tumor necrosis factor-α; IL-1β, interleukin-1β; IgG, immunoglobulin G; IgA, immunoglobulin A; IgM, immunoglobulin M; SOD, superoxide dismutase; MDA, malondialdehyde; T-AOC, total antioxidant capacity; CAT, catalase.

**Table 5:** Effects of FPKC on rumen fermentation parameters of growing–fattening beef cattle.

Items	Treatments				*SEM*	*p*-Value		
	CON	10% FPKC	20% FPKC	30% FPKC		*Treatment Effect*	*Linear Effect*	*Quadratic Effect*
pH	6.69	6.67	6.45	6.57	0.055	0.407	0.240	0.432
MCP, mg/mL	1.29 ^b^	1.30 ^b^	1.41 ^a^	1.30 ^b^	0.018	0.039	0.359	0.186
NH3-N, mg/dL	16.92	16.20	15.15	15.89	0.055	0.761	0.426	0.599
TVFA, mmol/L	51.00	50.89	51.63	50.59	0.396	0.855	0.898	0.853
Acetate, mmol/L	35.80	36.19	36.66	36.22	0.184	0.469	0.310	0.323
Propionate, mmol/L	7.42 ^C^	7.86 ^B^	8.35 ^A^	7.84 ^B^	0.098	0.001	0.040	0.001
Acetate/propionate, mmol/L	4.83 ^a^	4.61 ^ab^	4.39 ^b^	4.62 ^ab^	0.056	0.030	0.094	0.018
Butyrate, mmol/L	6.31	5.52	5.47	5.35	0.275	0.635	0.242	0.433
Valerate, mmol/L	0.27	0.28	0.25	0.27	0.015	0.942	0.841	0.889
Isobutyrate, mmol/L	0.40	0.36	0.28	0.32	0.025	0.373	0.157	0.279
Isovalerate, mmol/L	0.80	0.69	0.60	0.60	0.039	0.275	0.052	0.132

a,b: Within a row, values without a common superscript differ significantly (*p* < 0.05). A–C: Within a row, there are highly significant differences (*p* < 0.01) between values without a common superscript.

## Data Availability

The 16S rRNA gene sequencing data were deposited in the NCBI BioProject database under accession number PRJNA1405929. Other datasets employed and analyzed in this study are obtainable from the corresponding author upon reasonable request.

## Supplementary Materials

The following supporting information can be downloaded at: https://www.mdpi.com/article/10.3390/ani16142136/s1, Table S1: Effects of FPKC on rumen microbial alpha diversity indices of growing-fattening beef cattle; Table S2: Relative abundance (%) of rumen microbiota in growing-fattening beef cattle at the phylum level (relative abundance > 1% in at least one treatment); Table S3: Relative abundance (%) of rumen microbiota in growing-fattening beef cattle at the genus level (relative abundance > 1% in at least one treatment).
