# Supplementary material for: Effects of Fermented Palm Kernel Cake on Growth Performance, Serum Indices, and Rumen Microbiota in Growing–Fattening Beef Cattle
Source: Animals (Basel). 2026 Jul 9;16(14):2136. doi: 10.3390/ani16142136 (PMC13403445; doi:10.3390/ani16142136)
Supplement: Supplementary file 1 [file animals-16-02136-s001.zip › animals-4301879-supplementary.pdf]

Table S1. Effects of FPKC on rumen microbial alpha diversity indices of growing-fattening beef cattle.

Items	Treatments				*SEM*	*p*-value
	CON	10%FPKC	20%FPKC	30%FPKC		
Sobs	1223.50	1183.50	1142.50	1171.75	17.730	0.482
Shannon	6.04	5.83	5.42	5.69	0.079	0.066
Simpson	0.006	0.009	0.022	0.013	0.002	0.057
ACE	1257.80	1227.54	1209.72	1220.33	15.565	0.765
Chao1	1269.34	1245.54	1225.77	1236.04	15.459	0.816

Table S2. Relative abundance (%) of rumen microbiota in growing-fattening beef cattle at the phylum level (relative abundance > 1% in at least one treatment).

Items	Treatments				*SEM*	*p*-value
	CON	10%FPKC	20%FPKC	30%FPKC		
Bacteroidota	51.74	43.52	46.54	53.28	1.548	0.073
Bacillota	40.50	42.80	29.33	34.40	2.236	0.123
Proteobacteria	1.84^{b}	6.97^{b}	19.31^{a}	7.40^{b}	2.325	0.032
Patescibacteria	2.78	3.29	1.96	2.00	0.233	0.112
Verrucomicrobiota	1.75	1.89	1.72	1.30	0.207	0.805

Table S3. Relative abundance (%) of rumen microbiota in growing-fattening beef cattle at the genus level (relative abundance > 1% in at least one treatment).

Phylum level	Genus level	Treatments				*SEM*	*p*-value
		CON	10%FPKC	20%FPKC	30%FPKC		
Bacteroidota							
	Rikenellaceae_RC9_gut_group	11.39	11.93	10.40	13.90	1.021	0.712
	norank_f__F082	6.81^{a}	4.47^{bc}	3.28^{c}	5.64^{ab}	0.446	0.013
	norank_o__RF39	3.25	2.70	2.59	2.17	0.228	0.445
	norank_f__Muribaculaceae	2.23^{ab}	2.76^{a}	0.96^{b}	0.97^{b}	0.296	0.047
	norank_o__WCHB1-41	1.71	1.88	1.71	1.25	0.208	0.782
	Prevotellaceae_UCG-001	1.52	1.38	1.59	1.31	0.150	0.930
	Prevotellaceae_UCG-003	1.49	1.21	0.82	1.08	0.124	0.300
	norank_f__p-251-o5	1.30	1.20	0.40	0.62	0.180	0.220
Bacillota							
	Xylanibacter	19.38	16.27	23.89	24.80	1.637	0.222
	norank_o__Clostridia_UCG-014	5.06^{a}	6.03^{a}	4.46^{ab}	2.69^{b}	0.437	0.028
	NK4A214_group	4.03	3.05	0.76	3.09	0.514	0.127
	Ruminococcus	2.10	3.45	2.35	2.35	0.320	0.487
	norank_f__UCG-011	2.88^{A}	2.64^{A}	1.09^{B}	3.11^{A}	0.256	0.006

	Christensenellaceae_R-7_group	3.10[a]	2.63[a]	0.81[b]	2.61[a]	0.328	0.045
	norank_f__[Eubacterium]_coprostanoligenes_group	1.49	1.81	1.71	1.65	0.126	0.874
	Succiniclasticum	1.79	2.18	0.90	1.22	0.192	0.065
	norank_f__UCG-010	1.19	1.24	0.73	1.13	0.096	0.234
	Butyrivibrio	1.08	1.31	0.72	1.14	0.103	0.234
	Pseudobutyrivibrio	0.62	1.07	1.35	1.15	0.162	0.465
	unclassified_f__Lachnospiraceae	0.39	0.71	1.32	1.21	0.228	0.481
Proteobacteria							
	Succinivibrionaceae_UCG-002	1.08[b]	4.70[b]	13.61[a]	5.86[ab]	1.703	0.043
	Candidatus_Saccharimonas	2.37	2.58	1.56	1.66	0.208	0.222
